# Intravital imaging of mucus transport in asthmatic mice using microscopic optical coherence tomography

**DOI:** 10.1152/ajplung.00455.2021

**Published:** 2022-08-23

**Authors:** Mario Pieper, Hinnerk Schulz-Hildebrandt, Inken Schmudde, Katharina M. Quell, Yves Laumonnier, Gereon Hüttmann, Peter König

**Affiliations:** ^1^Institute of Anatomy, University of Lübeck, Lübeck, Germany; ^2^Airway Research Center North (ARCN), University of Lübeck, German Center for Lung Research (DZL), Lübeck, Germany; ^3^Institute of Biomedical Optics, University of Lübeck, Lübeck, Germany; ^4^Institute for Systemic Inflammation Research, University of Lübeck, Lübeck, Germany; ^5^Center of Brain, Behavior and Metabolism (CBBM), University of Lübeck, Lübeck, Germany

**Keywords:** asthmatic mice, hypertonic saline, mucus transport, OCT, trachea

## Abstract

Asthma is one of the most common chronic diseases. Mucus overproduction is consistently linked to asthma morbidity and mortality. Despite the knowledge of the importance of mucus, little data exist on how mucus is transported in asthma and the immediate effects of therapeutic intervention. We therefore used microscopic optical coherence tomography (mOCT) to study spontaneous and induced mucus transport in an interleukin-13 (IL-13)-induced asthma mouse model and examined the effects of isotonic (0.9% NaCl) and hypertonic saline (7% NaCl), which are used to induce mucus transport in cystic fibrosis. Without intervention, no bulk mucus transport was observed by mOCT and no intraluminal mucus was detectable in the intrapulmonary airways by histology. Administration of ATP-γ-S induced mucus secretion into the airway lumen, but it did not result in bulk mucus transport in the trachea. Intraluminal-secreted immobile mucus could be mobilized by administration of isotonic or hypertonic saline but hypertonic saline mobilized mucus more reliably than isotonic saline. Irrespective of saline concentration, the mucus was transported in mucus chunks. In contrast to isotonic saline solution, hypertonic saline solution alone was able to induce mucus secretion. In conclusion, mOCT is suitable to examine the effects of mucus-mobilizing therapies in vivo. Although hypertonic saline was more efficient in inducing mucus transport, it induced mucus secretion, which might explain its limited benefit in patients with asthma.

## Graphical abstract

**Figure:**
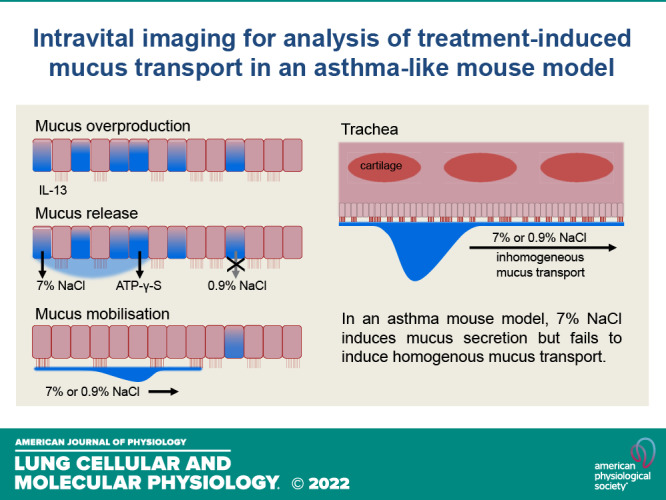

## INTRODUCTION

Asthma is a heterogeneous chronic inflammatory respiratory disease that leads to airway obstruction. Although symptoms can generally be treated well, asthma exacerbation causes major disease burden for patients (1). Particularly in severe asthma, symptoms are often accompanied by an increased amount of intraluminal mucus in the airways that can lead to restriction of airflow (2–4). Thus, the removal of mucus and the inhibition of mucus release represent important therapeutic concepts for asthma (5, 6). However, to date, no licensed drug is directly addressing mucus transport or prevention of release in patients with asthma, and more research in this area is needed (7).

One of the obstacles in mucus research is the lack of techniques that allow direct visualization of mucus transport to understand the immediate effects of individual therapeutic interventions. We recently developed a method to study mucus transport and the effects of isotonic and hypertonic saline on mucus transport in living mice by high-speed microscopic optical coherence tomography (mOCT) in *Scnn1b*-transgenic mice (8), which serve as a model of chronic mucoobstructive disease resembling cystic fibrosis (9).

For assessing mucus transport, mOCT is well suited because it relies on reflected light from the tissue to generate contrast and can visualize airway structure and mucus without external contrast agents (10). It reaches frame rates above 80 Hz and provides cross-sectional images with a spatial resolution of less than 1.5 µm. Furthermore, mOCT can be used for imaging over extended periods without harming the tissue (11).

In bronchial asthma, IL-13 plays a key role. Administration of IL-13 in mice is sufficient to induce airway hyperreactivity and metaplasia of airway epithelial cells toward a goblet cell phenotype that can secret viscous mucus. Both are central features of asthma. Consequently, blocking of IL-13 signaling was successful in preventing asthma symptoms in animal models (12, 13). Recently, antibodies blocking the IL4/IL-13 receptor were successfully established as a treatment for a subtype of asthma (14). The mucus induced by IL-13 administration predominantly contains the mucin Muc5AC that is the predominant mucin found in asthmatic mucus (15). Therefore, intratracheal administration of IL-13 in mice appears to be a fast and reliable model to study asthma-related mucus transport. We used the IL-13 mouse model in combination with mOCT to study mucus transport and assessed the effects of isotonic and hypertonic saline and evaluated if mOCT is a suitable method to study mucus transport in mouse models of asthma.

## METHODS

### Animals

In all imaging experiments, BALB/cAnNCrl mice (Charles River Laboratories, Sulzfeld, Germany) aged 6–8 wk were used. Animal care was provided in accordance with German law and the study was approved by the Schleswig-Holstein state authorities (V 242–63561/2017).

### Induction of Asthma-Like Airway Disease

For induction of airway disease, mice were anesthetized with 45 mg/kg body wt ketamine + 5.5 mg/kg body wt xylazine in 350 µL intraperitoneally on *days 0, 1*, and *2*, and mice received 10-µg mouse IL-13 (BioLegend, San Diego, CA) dissolved in 50-µL PBS intratracheally. Control mice received 50-µL PBS.

### Optical Coherence Microscopy System and Image Acquisition

A detailed description of the mOCT setup optimized for intravital imaging can be found in previous publications (8, 16). In brief, light from a low noise super-continuum source (EXW-4 OCT, NKT Photonics, Birkerød, Denmark) is split by a fiber beam splitter (TW670R5A2, Thorlabs GmbH, Bergkirchen, Germany) with splitting ratio of 50:50 into the reference and sample arm of the Michelson interferometer. In the reference arm, the light is collimated (fiber collimator 60FC, Schäfter + Kirchhoff, Hamburg, Germany). After passing through a variable neutral density filter and an SF57 glass block to precompensate for dispersion, the light is reflected back by a prism (PS975M, Thorlabs GmbH). In the sample arm, the collimated light is deflected by a pair of galvanometer scanners (6210H, Cambridge Technology, Garching, Germany). Both scanner mirrors are imaged on each other by 4f-optics. After beam expansion with a telescope (TTL200MP and SL50-CLS2, Thorlabs GmbH, Bergkirchen, Germany), the light is focused into the sample via a ×5/0.16 NA objective (EC Plan-Neofluar, Carl Zeiss, Oberkochen, Germany).

The light backscattered from the reference and sample arm is recombined in the fiber beam splitter and brought to interference on a customized high-speed spectrometer (Thorlabs GmbH, Bergkirchen, Germany). The 2,048 pixel spectrometer covers a wavelength range from 570 nm to 930 nm. Measurements were done with an A-scan rate of up to 250 kHz and a B-scan rate of 80 Hz. Acquired spectra were resampled, Hann windowed, and Fourier transformed to obtain the OCT A-scans. Remaining dispersion of higher order was numerically corrected in postprocessing (17).

### In Vivo Imaging

Imaging mOCT system was performed according to the protocol of our previous study on mucus transport in muco-obstructive lung disease (8). Anesthesia of mice was achieved by intraperitoneal injection of 300 µL of MMF [0.48 mg/mL midazolam (AlleMan Pharma GmbH, Rimbach, Germany), 48 µg/mL medetomidine (Pfizer, Berlin, Germany), and 12 µg/mL fentanyl (Janssen, Neuss, Germany)] in 0.9% NaCl. The mice were placed on a tempered intravital stage (37°C), which was adjustable in all directions. The trachea was exposed by removing the overlying skin, displacing the submandibular glands, and removing the infrahyoid musculature. For imaging through the intact trachea, the mouse was directly positioned under the microscope objective. Mucus transport was visualized in a field of view of 4 mm by using a ×5/0.16 NA objective (EC Plan-Neofluar, Carl Zeiss, Oberkochen, Germany). Here, a lateral resolution of 1.8 µm was achieved. Images were acquired in a sequence of 5 frames at 80 Hz, which were repeated every second. The baseline mucus thickness was measured for 10 min before the interventions. To stimulate mucus secretion in the lungs, we nebulized 10 µM ATP-γ-S (Abcam, Cambridge, UK) in 0.9% NaCl or only 0.9% NaCl as vehicle for 10 s with a PARI BOY SX (PARI GmbH, Starnberg, Germany) that was positioned in front of the snout. After 30 min, 30 µL 0.9% NaCl and 7% NaCl solution were applied intranasally. Changes of mucus thickness in the trachea were imaged over 60 min. In total four groups of animals treated with ATP-γ-S and four control groups were imaged and evaluated (Table 1).

**Table 1.** Overview over experimental groups

	Pretreatment	Mucus Release	Mucus Mobilization
Group 1	Control	Nebulized vehicle	Intranasal 0.9% NaCl
Group 2	Control	Nebulized ATP-γ-S	
Group 3	IL-13 treatment	Nebulized vehicle	Intranasal 0.9% NaCl
Group 4	IL-13 treatment	Nebulized ATP-γ-S	Intranasal 0.9% NaCl
Group 5	Control	Nebulized vehicle	Intranasal 7% NaCl
Group 6	Control	Nebulized ATP-γ-S	Intranasal 7% NaCl
Group 7	IL-13 treatment	Nebulized vehicle	Intranasal 7% NaCl
Group 8	IL-13 treatment	Nebulized ATP-γ-S	Intranasal 7% NaCl

Control = PBS treatment; vehicle = 0.9% NaCl.

### Lung Function Measurements

Airway responses were evaluated using a flexiVent system (SCIREQ Scientific Respiratory Equipment Inc, Montreal, Canada). Mice were anesthetized by intraperitoneal injection of 300 µL of 0.48 mg/mL midazolam (AlleMan Pharma GmbH, Rimbach, Germany), 48 µg/mL medetomidine (Pfizer, Berlin, Germany), and 12 µg/mL fentanyl (Janssen, Neuss, Germany) in 0.9% NaCl (MMF). Mouse tracheas were cannulated with a 20-gauge blunt needle and the mice were ventilated at 150 breaths/min. Mice were paralyzed with pancuronium bromide (0.8 mg/kg) and allowed to stabilize on the ventilator for 5 min. Mice were then exposed to aerosolized methacholine (0, 2, 5, 10, 25, and 50 mg/mL) in isotonic saline for 15 s using an ultrasonic nebulizer (Aeroneb, Ratingen, Germany) and ventilated for additional 10 s. Ventilation cycle measurements were made until resistance peaked, then airways were rerecruited with deep inflation and the next higher dose was applied.

### Histology

After the in vivo experiment, mice were euthanized, the chest was opened, and the lungs were filled with 4% (wt/vol) phosphate-buffered paraformaldehyde with a pressure of 25 cmH_2_O via a tracheal cannula and the trachea was subsequently ligated to prevent leakage of the fixative. The lungs were removed from the thoracal cavity and immersed in 4% paraformaldehyde overnight and embedded in paraffin. To assess the presence of mucus in the airways of the lungs, 5-µm-thick lung sections were stained with Alcian blue and periodic acid-Schiff reagent (AB-PAS). To compare the amount of mucus in the lungs, the area of mucus in the large airways was measured in the histological sections using Matlab (MATLAB R2019b, The MathWorks, Inc.). The measured area was multiplied by the section thickness and the calculated volume was related to the epithelial surface of the airway. The surface area included in this calculation was determined by multiplying the circumference of the airway lumen by the section thickness.

### Measurement of Mucus Parameters and Statistical Analysis

Every minute, we determined the changes in mucus layer thickness after stimulation by measuring the distance between the highly scattering subepithelial fibers and the highly reflective air-liquid interface at four different points of the trachea. The mean value of all four points was calculated for each minute and the mean thickness before stimulation was subtracted from the subsequent measurements. To get measure for the uniformity of the mucus, we calculated the mucus thickness variance. The four measurements of mucus layer thickness that were used to calculate mean mucus thickness were normalized to the highest value and then the variance was calculated. The mean of the variance throughout the observation period was calculated for each mouse as described in reference (8).

Two different transport speeds were measured. A more constant velocity of the mucus most likely due to ciliary beating was calculated by measuring the distance of particles near the epithelium over two frames and dividing by the frame time. In addition, the velocity of mucus chunks, which are most likely transported by a combination of ciliary beating, respiration, and cough-like events, was calculated by measuring the distance between two mucus hilltops between two frames.

Statistical comparisons between two groups were performed by Mann–Whitney *U* test to detect differences in airway hyperresponsiveness measurement. We used the Wilcoxon signed-rank test to compare the related values for mucus thickness changes after mucus release and subsequent mucus mobilization treatment. The results were regarded as statistically significant if *P* < 0.05.

## RESULTS

### IL-13 Induces Airway Hyperresponsiveness and Goblet Cell Metaplasia

**Figure 1.**
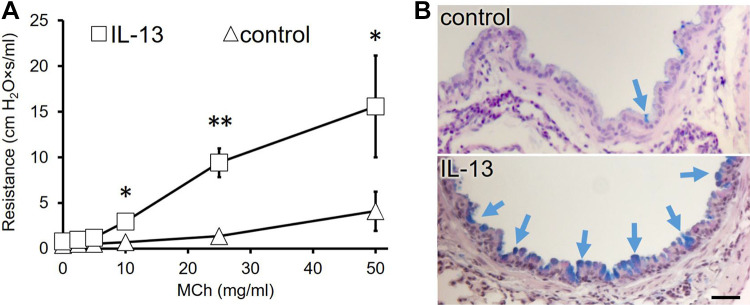
IL-13 induces airway hyperresponsiveness and goblet cell metaplasia. *A*: provocation with increasing concentrations of metacholine (MCh) induced higher airway resistance in IL-13-treated mice than in PBS-treated control mice. Data are presented as means ± SE (*n* = 5 mice), *P < 0.05, **P < 0.01. *B*: AB-PAS staining in lung sections demonstrates more AB-PAS-positive epithelial cells (blue arrows) in lungs from IL-13-treated mice compared with control mice. Scale bar, 20 μm.

To confirm that IL-13 in our hands induces the main features of asthma in mice, we determined the airway resistance in response to escalating nebulized doses of methacholine. As expected, mice treated with IL-13 showed increased airway resistance (Fig. 1*A*). AB-PAS staining of lungs confirmed that mice treated with IL-13 alone exhibited higher numbers of AB-PAS-positive mucus-producing airway epithelial cells (Fig. 1*B*).

### ATP-γ-S Induces Mucus Secretion but No Bulk Mucus Transport

**Figure 2.**
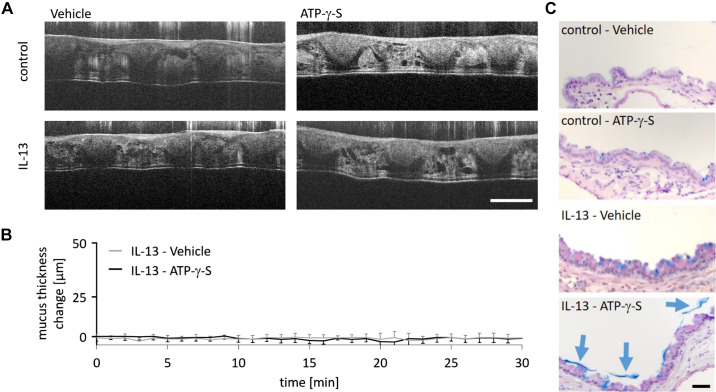
ATP-γ-S induces mucus secretion into the airway lumen without further transport in IL-13-treated mice. *A*: intravital mOCT imaging of the trachea demonstrated only a very thin mucus layer with no mucus transport in control and IL-13-treated mice after vehicle or ATP-γ-S nebulization (see also Supplemental Video S1). Scale bar, 200 µm. *B*: quantification of mucus thickness change in IL-13-treated mice over 30 min after vehicle or ATP-γ-S nebulization. Data are presented as means ± SE (*n* = 6 or 7 mice). *C*: AB-PAS staining of section from the lungs harvested after the imaging experiments. Intraluminal mucus (blue arrows) was detectable in ATP-γ-S-treated animals. Scale bar, 20 µm. AB-PAS, Alcian blue and periodic acid-Schiff reagent; mOCT, microscopic optical coherence tomography.

In mice treated with IL-13, we did not observe bulk mucus transport without further intervention (not shown). In addition, we could barely detect any mucus in the airway lumen in these mice by histology indicating that only basal amounts of mucus are present without stimulation of mucus release. To induce mucus secretion, we exposed IL-13-treated and control mice to nebulized ATP-γ-S, which is a potent secretagogue, and compared it to mice that received nebulized vehicle. Irrespective of the nebulized substance, we observed only a thin layer of mucus in the tracheas and no bulk mucus transport in all mice examined using mOCT (Fig. 2, *A* and *B*, and Supplemental Video S1; see https://doi.org/10.6084/m9.figshare.16940371).

To verify that ATP-γ-S induced mucus release, we stained histological sections of the lungs with AB-PAS (Fig. 2*C* and Supplemental Fig. S1; see https://doi.org/10.6084/m9.figshare.19825825.v1). In mice treated with IL-13 followed by nebulization with ATP-γ-S, we observed mucus sheets in the airway lumen. No intraluminal mucus was detectable in animals that were treated with IL-13 followed by nebulization of vehicle although mucus was readily detectable in airway epithelial cells. No intraluminal mucus and only very few AB-PAS-positive cells were detectable in control mice that received ATP-γ-S or vehicle. These observations confirm that ATP-γ-S induced mucus release in IL-13-treated mice but the mucus was not efficiently transported to the trachea within the observation period.

### Isotonic Saline Mobilizes Secreted Mucus from the Lungs

**Figure 3.**
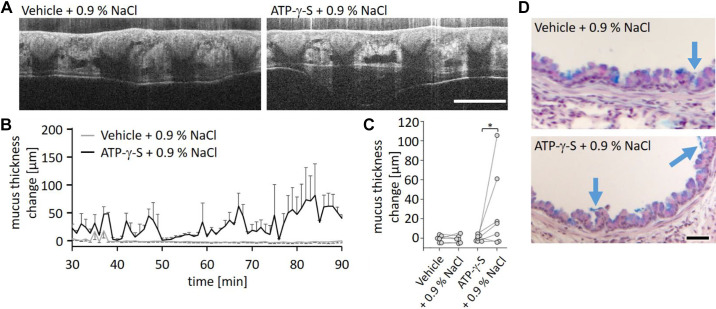
Intranasal administration of isotonic saline following ATP-γ-S nebulization leads to mucus transport in IL-13-treated mice. *A*: intravital mOCT imaging of the trachea of IL-13-treated mice. Intranasal isotonic saline administration 30 min after nebulization of vehicle did not lead to visible mucus transport (see also Supplemental Video S3). Nebulization of ATP-γ-S followed by intranasal administration of isotonic saline lead to mucus transport (see Supplemental Video S2). Scale bar, 200 µm. *B*: measurement of mucus thickness change in IL-13-treated mice over 60 min after intranasal administration of isotonic saline with prior nebulization of vehicle or ATP-γ-S. Data are presented as means ± SE (*n* = 6 or 7 mice). *C*: change of mucus thickness averaged over 60 min for each mouse (represented by a circle) after nebulization of vehicle or ATP-γ-S. The associated values of each mouse before and after administration of 0.9% NaCl are connected. *P < 0.05. *D*: AB-PAS staining of lungs harvested after imaging experiments. In mice intranasally treated with isotonic saline after ATP-γ-S nebulization or vehicle, mucus residues (blue arrows) were present on top of the epithelium. Scale bar, 20 µm. AB-PAS, Alcian blue and periodic acid-Schiff reagent; mOCT, microscopic optical coherence tomography.

We next examined if treatment with isotonic saline was able to induce transport of the secreted mucus in mice treated with IL-13 and ATP-γ-S. Intranasal application of isotonic saline induced mucus transport (Fig. 3, *A* and *B*, and Supplemental Video S2; see https://doi.org/10.6084/m9.figshare.16940380). In contrast, all IL-13-treated mice that received only nebulized vehicle and subsequent treatment with intranasal isotonic saline showed no mucus transport (Fig. 3, *A* and *C*, and Supplemental Video S3; see https://doi.org/10.6084/m9.figshare.16940377). After ATP-γ-S nebulization with subsequent administration of 0.9% NaCl, we detected a significant mucus transport after 0.9% NaCl administration although the response in individual mice varied (Fig. 3*C*). AB-PAS staining of lung sections from these mice showed only small residual intraluminal mucus (Fig. 3*D*) and overview images and quantification of mucus amounts show that the mucus released by ATP-γ-S was mostly removed from the lungs (Supplemental Fig. S1).

### Hypertonic Saline Induces Mucus Secretion and Mucus Transport

**Figure 4.**
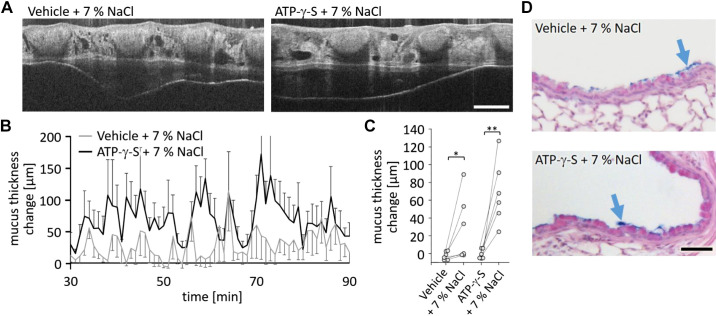
Intranasal administration of hypertonic saline leads to mucus transport irrespective of nebulization of ATP-γ-S or vehicle in IL-13-treated mice. *A*: intravital mOCT imaging of the trachea of mice demonstrates mucus transport after intranasal administration of hypertonic saline following nebulization of vehicle or ATP-γ-S (see Supplemental Videos S4 and S5). Scale bar, 200 µm. *B*: measurement of mucus thickness change in IL-13-treated mice over 60 min after intranasal administration of hypertonic saline with prior nebulization of vehicle or ATP-γ-S. Data are presented as means ± SE (*n* = 6 or 7 mice). *C*: change of mucus thickness averaged over 60 min for each mouse after nebulization of vehicle or ATP-γ-S. The associated values are connected with the values measured for the same mouse after additional administration of 7% NaCl. *P < 0.05, **P < 0.01. *D*: AB-PAS staining of sections from lungs dissected after imaging experiments. Following intranasal administration of hypertonic saline, intraluminal mucus (blue arrows) was detectable in mice with prior nebulization of vehicle or ATP-γ-S. Scale bar, 20 µm. AB-PAS, Alcian blue and periodic acid-Schiff reagent; mOCT, microscopic optical coherence tomography.

We have previously shown in *Scnn1b*-transgenic mice that hypertonic saline was able to induce more effective mucus transport compared with isotonic saline. In IL-13-treated mice that received nebulized vehicle, we found that intranasal administration of hypertonic saline in contrast to isotonic saline induced significant mucus transport without prior induction of mucus secretion (Fig. 4, *A* and *B*, and Supplemental Video S4; see https://doi.org/10.6084/m9.figshare.16940374). After administration of 7% NaCl, 3 out of 6 mice show clear transport and increased mucus thickness (Fig. 4*C*). Lung sections stained with AB-PAS confirmed that hypertonic saline alone was able to induce mucus secretion (Fig. 4*D*). This indicates that hypertonic saline is able to induce mucus release from goblet cells. In IL-13-treated mice that had received ATP-γ-S, hypertonic saline induced substantial mucus transport in all 6 mice (Fig. 4, *A*–*C*, and Supplemental Video S5; see https://doi.org/10.6084/m9.figshare.16940368). Although hypertonic saline following ATP-γ-S application-induced substantial mucus transport in all mice, we still detected small amounts of intraluminal secreted mucus by histology (Fig. 4*D*). However, mucus quantifications and overview images of the lungs sections show that the mucus released by ATP-γ-S was removed by the therapeutic intervention with hypertonic saline solution (Supplemental Fig. S1).

### Mucus Transport in the Asthma Model Is Nonuniform

**Figure 5.**
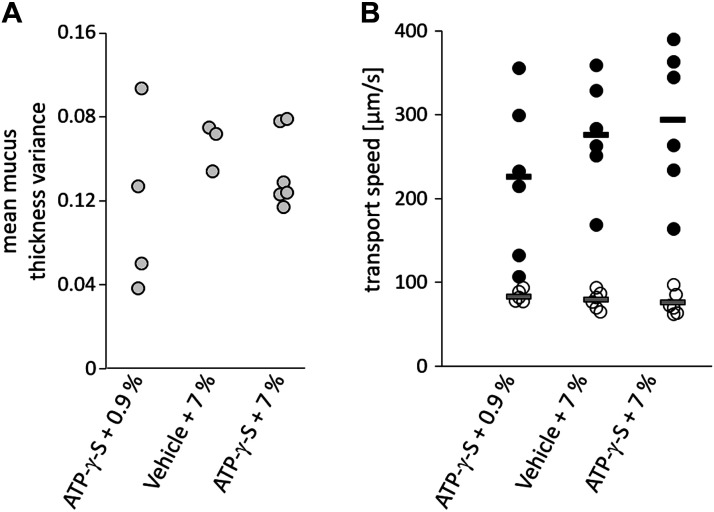
Mucus thickness variance and transport velocity. *A*: as an indicator for the homogeneity of the transport, we calculated the mean mucus thickness variance for the mice that showed a clear transport with a mean mucus thickness change of more than 10 µm in 60 min. Each circle represents one mouse analyzed. *B*: two different mucus velocities where measured. The open circles represent the particle speed near the epithelium (ciliary-mediated transport). The filled circles represent the speed of the mucus chunks mediated by cilia beating, respiration, and cough-like events. The bars correspond to the mean values of the respective velocities.

To determine the mucus height homogeneity as an indicator of uniform mucus distribution, we calculated the mean variance of mucus thickness in the mice that showed a clear transport with a mean mucus thickness change of more than 10 µm in 60 min. Mean variance of mucus thickness did not depend on the concentration of the NaCl solution (Fig. 5*A*), indicating that mucus mobilized by hypertonic saline was not more evenly distributed on the surface of the epithelium, an effect of hypertonic saline we have previously observed in *Scnn1b*-transgenic mice. Analysis of the particle transport near the epithelium of these mice revealed a mean velocity below 100 µm/s. The transport speed of mucus chunks varied considerably among animals, ranging from 107 µm/s to 391 µm/s. Overall, no significant differences in transport velocities were determined for the different treatments (Fig. 5*B*).

## DISCUSSION

Mucus hypersecretion and mucus plugging are major problems in asthma. Although there is a clinical need, no therapeutic approach that specifically targets mucus in asthmatic disease is clinically approved or in the process of application. Using mOCT, we were able to monitor mucus transport in a mouse model of allergic asthma thereby opening up the possibility to test novel therapeutic approaches.

A major observation from our study is that in the mouse model we used, no bulk mucus transport was detected without further intervention, and accordingly, no increased amounts of mucus were detectable by histology. As the air-liquid interface produces a very strong signal and we are limited in the axial resolution, we cannot resolve a thin basal mucus layer by imaging through the tracheal wall in living mice. Our observations do not exclude release and transport of a thin native mucus layer (18). Consequently, application of isotonic saline did not induce increased mucus transport. This is in contrast to our previous study with *Scnn1b*-transgenic mice, in which isotonic saline reliably induced bulk mucus transport (8). This can be explained by the fact that *Scnn1b*-transgenic mice harbor significant amounts of mucus in the airway lumen without stimulation of mucus secretion (19).

In contrast, in IL-13-treated mice, metaplasia of the airway epithelium toward goblet cells was observed and mucus was readily detectable in these cells but our data confirm that this mucus was not spontaneously released by handling, anesthesia, or imaging. To stimulate mucus release, a process that is expected during asthma exacerbation, we applied the secretagogue ATP-γ-S. This approach led to mucus secretion as judged by histological examination of the treated lungs. Despite readily detectable mucus in the airways, no bulk mucus transport was detectable in the trachea. This demonstrates that the mucus released was not efficiently transported by mucociliary clearance, which is in accordance with the observation that the mucus in patients with asthma is not readily transported (20).

Administration of hypertonic saline after application of ATP-γ-S robustly induced mucus transport in all IL-13-treated animals and was more efficient than isotonic saline to mobilize mucus. This increased ability of hypertonic saline compared with isotonic saline to mobilize mucus was also observed in *Scnn1b*-transgenic mice. However, it is not clear if the ability of hypertonic saline to mobilize mucus is solely due to increased mobilization of intraluminal mucus or if hypertonic saline also induces mucus secretion. Our data support the notion that hypertonic saline itself is able to induce mucus secretion in IL-13-treated mice since we observed bulk mucus transport in the trachea in 50% of mice treated with hypertonic saline without prior application of ATP-γ-S. The ability of hypertonic saline to induce mucus secretion could in part explain the mediocre clinical efficiency in patients with asthma compared with their success in the therapy of patients with cystic fibrosis (21).

In *Scnn1b*-transgenic mice, we previously observed that the mucus mobilized by hypertonic saline was different from mucus mobilized by isotonic saline. Mucus mobilized by hypertonic saline was more evenly distributed over the airway epithelium compared with mucus that was mobilized by isotonic saline, which was mainly transported as mucus chunks. We did not observe the same effect in the IL-13 model in which both isotonic and hypertonic saline induced mucus transport in the form of chunks.

We also quantified the chunkiness of mucus transport by calculating the variance of mucus height at individual time points and calculated the mean variance over the observation period. If mucus is evenly distributed, the mucus height in all points is comparable and the variance is low. If mucus is transported in chunks, the mucus height in some points is larger than in others and the variance is higher. By calculating the mean variance over the observation time, it is possible to quantify the general chunkiness of transported mucus. Consequently, in *Scnn1b*-transgenic mice treated with hypertonic saline, the mean variance was lower than mice treated with isotonic saline (8). In accordance with the observation that hypertonic saline failed to change mucus properties in mice treated with IL-13, we measured a similar variance between mucus mobilized by isotonic and hypertonic saline. Furthermore, the mean variance of mucus released by hypertonic saline in IL-13-treated mice was higher than in *Scnn1b*-transgenic mice. The fact that hypertonic saline induces a uniform transport in the *Scnn1b*-transgenic mice and a nonuniform transport in the IL-13 model suggests different mucus compositions. Although we did not measure the composition of the mucins in our models ourselves, evidence for different compositions is found in the literature. The mucus in IL-13-treated mice and in patients with asthma contains a high proportion of the mucin Muc5AC (22). Whereas in *Scnn1b*-transgenic mice, the mucus predominantly contains Muc5B (23). Muc5AC is known to increase mucus viscoelasticity and is associated with exacerbation risk in severe asthma (24, 25). In addition, it was observed that Muc5AC is attached to the cell surface further hampering efficient transport (26). Thus, the reason for the lack of mucus transport after ATP-γ-S nebulization and the inability of hypertonic saline to influence reduction in the chunkiness of the mucus could be attributed to the high Muc5AC content. It is possible that the airway surface liquid present was not sufficient to properly hydrate the secreted mucus that requires water to fully expand (27) resulting in a sticky mucus in IL-13 mice. Therefore, it is also reasonable to assume that the difference between IL-13 and Scnn1b is because different thresholds of hydration are required in IL-13-treated mice compared with Scnn1b-transgenic mice to restore normal, nonheterogeneous mucus transport.

Our data lead to several insights into mucus transport in the IL-13 model of allergic airway disease. First, no mucus plugs are present in the airways without prior stimulation of mucus release. Second, stimulation of mucus secretion by ATP-γ-S leads to mucus secretion that is not efficiently transported. Third, the mucus secreted is distinct from mucus that is present in the airways of *Scnn1b*-transgenic mice.

Based on these observations we conclude that the IL-13-based mouse model is suitable to study therapeutic interventions that aim to mobilize intraluminal mucus but can also be used to assess strategies that prevent mucus release from goblet cells. Mucus is a clinical problem in patients with asthma (4), and new therapeutic strategies specifically addressing mucus in asthma are needed. The use of this model could speed up the early stages of new drug development specifically targeting mucus release and transport in asthma.

## DATA AVAILABILITY

Data will be made available upon reasonable request.

## SUPPLEMENTAL DATA

10.6084/m9.figshare.16940371 Supplemental Video S1: https://doi.org/10.6084/m9.figshare.16940371.

10.6084/m9.figshare.16940380 Supplemental Video S2: https://doi.org/10.6084/m9.figshare.16940380.

10.6084/m9.figshare.16940377 Supplemental Video S3: https://doi.org/10.6084/m9.figshare.16940377.

10.6084/m9.figshare.16940374 Supplemental Video S4: https://doi.org/10.6084/m9.figshare.16940374.

10.6084/m9.figshare.16940368 Supplemental Video S5: https://doi.org/10.6084/m9.figshare.16940368.

10.6084/m9.figshare.19825825.v1 Supplemental Fig. S1: https://doi.org/10.6084/m9.figshare.19825825.v1.

## GRANTS

This study was supported by the German Federal Ministry of Education and Research (82DZL001B2).

## DISCLOSURES

No conflicts of interest, financial or otherwise, are declared by the authors.

## AUTHOR CONTRIBUTIONS

M.P., H.S.-H., and P.K. conceived and designed research; M.P., I.S., and K.M.Q. performed experiments; M.P. and H.S.-H. analyzed data; M.P. and P.K. interpreted results of experiments; M.P. prepared figures; M.P. drafted manuscript; M.P., H.S.-H., I.S., Y.L., G.H., and P.K. edited and revised manuscript; M.P., H.S.-H., I.S., K.M.Q., Y.L., G.H. and P.K. approved final version of manuscript.
